# A role for annexin A2 in scaffolding the peroxiredoxin 2–STAT3 redox relay complex

**DOI:** 10.1038/s41467-020-18324-9

**Published:** 2020-09-09

**Authors:** Deepti Talwar, Joris Messens, Tobias P. Dick

**Affiliations:** 1 Division of Redox Regulation, DKFZ-ZMBH Alliance, German Cancer Research Center (DKFZ), Im Neuenheimer Feld 280, 69120 Heidelberg, Germany; 2 Faculty of Biosciences, Heidelberg University, 69120 Heidelberg, Germany; 3 VIB-VUB Center for Structural Biology, Vlaams Instituut voor Biotechnologie, B-1050 Brussels, Belgium; 4 Brussels Center for Redox Biology, Vrije Universiteit Brussel, B-1050 Brussels, Belgium; 5 Structural Biology Brussels, Vrije Universiteit Brussel, B-1050 Brussels, Belgium

**Keywords:** Multienzyme complexes, Membrane proteins, Thioredoxins, Cell signalling

## Abstract

Hydrogen peroxide (H_2_O_2_) is recognized to act as a signaling molecule. Peroxiredoxins (Prxs) have the ability to transfer H_2_O_2_-derived oxidizing equivalents to redox-regulated target proteins, thus facilitating the transmission of H_2_O_2_ signals. It has remained unclear how Prxs and their target proteins are brought together to allow for target-specific protein thiol oxidation. Addressing the specific case of Prx2-dependent STAT3 oxidation, we here show that the association of the two proteins occurs prior to Prx oxidation and depends on a scaffolding protein, the membrane chaperone annexin A2. Deletion or depletion of annexin A2 interrupts the transfer of oxidizing equivalents from Prx2 to STAT3, which is observed to take place on membranes. These findings support the notion that the Prx2-STAT3 redox relay is part of a highly organized membrane signaling domain.

## Abstract (web summary)

Peroxiredoxin 2 (Prx2) was previously shown to transfer H_2_O_2_-derived oxidative equivalents to STAT3, generating disulfide-linked dimers and tetramers. Here the authors show that the interaction between Prx2 and STAT3 at the plasma membrane is mediated by the membrane chaperone annexin A2; suggesting that the redox relay complex is part of a membrane signaling domain.

## Introduction

Over the past few years, it has become clear that intracellular H_2_O_2_ can act as a signal by causing the oxidation of particular thiols on particular proteins. This led to the question of how H_2_O_2_ is able to oxidize individual protein thiols in a selective and efficient manner. We may not yet have the full picture, but we can now at least give partial answers to that question: Firstly, some redox-regulated proteins harbor thiols that can be directly oxidized by H_2_O_2_. These are proteins that have a primary function unrelated to H_2_O_2_ scavenging, but additionally evolved their own catalytic site to accelerate the reaction between H_2_O_2_ and one of their thiols. The two best-known examples are the bacterial transcription factor OxyR^1^ and the glycolytic enzyme GAPDH^2^. Secondly, it is conceivable that proteins lacking such built-in catalytic mechanisms are nevertheless directly oxidized by H_2_O_2_ when they are in immediate proximity to an H_2_O_2_ source, although it may turn out that H_2_O_2_ first needs to be converted into a different (intrinsically more reactive) kind of peroxide^3,4^. Thirdly, beyond direct oxidation, it has been recognized that some proteins can be oxidized indirectly, in a highly efficient and selective manner, through mediation by thiol peroxidases. The first known example was the Orp1-Yap1 redox relay discovered in yeast^5^. More recently, examples of peroxiredoxin-based redox relays were found in mammalian cells^6–8^, and there is emerging evidence for a role of cytosolic peroxiredoxins in transmitting oxidation to a multitude of target proteins^9^.

In principle, the existence of thiol peroxidase-based redox relays can answer a fundamental question: How can H_2_O_2_, barely larger than a water molecule, be specific in targeting particular thiols, when in fact the vast majority of protein thiols shows the same low H_2_O_2_ reactivity (*k* ≈ 1–10 M^−1^ s^−1^)^10^? It is hard to see how the tiny interaction surface between a H_2_O_2_ molecule and a protein surface can confer specificity towards specific sites for reaction. For a Prx-based redox relay the specificity question is much less of a problem: the Prx naturally acts as an ultra-efficient scavenger of H_2_O_2_ (*k* ≈ 10^5^–10^8^ M^−1^ s^−1^), and the specificity of target oxidation is then based on protein–protein interactions, namely those that bring together (directly or by involvement of additional proteins) the Prx and the target protein. Yet, although it is clear that protein–protein interaction can provide the necessary specificity, we still do not know how Prxs and their target proteins recognize each other and position themselves for productive oxidative transfer.

In this work, we asked three questions: Firstly, do Prxs and their target proteins co-assemble already in the reduced state, that is, are they pre-positioned even before the Prx reacts with H_2_O_2_? Secondly, which structural parts of the Prx and the target protein are required for the association to occur, and how specific is the interaction? Thirdly, do the two partners interact directly or is there a scaffold protein needed to template the interaction?

To address these questions, we focused on one specific example, namely the redox relay formed between peroxiredoxin 2 (Prx2) and the transcription factor STAT3 in human cells. Prx2 was previously shown to transfer H_2_O_2_-derived oxidative equivalents to STAT3, generating disulfide-linked dimers and tetramers of STAT3 which in turn attenuate transcription from the serum inducible element promoter^6^. The redox relay complex appears to be associated with the plasma membrane, as stimulation of the cell surface IL-6 receptor, presumably associated with NOX activation, triggers the oxidative transfer from Prx2 to STAT3^6^.

In this study, we show the following: (i) A non-covalent Prx2-STAT3 complex assembles independently of Prx2 oxidation, showing that the complex is already pre-formed in the reduced state and ready to respond to H_2_O_2_. (ii) Both proteins are highly selective towards each other: STAT3 strongly prefers Prx2 over the very similar isoform Prx1, and a single point mutation in STAT3 abolishes the interaction with Prx2. (iii) The interaction between Prx2 and STAT3 depends on a third protein, the membrane chaperone annexin A2 (AnxA2). (iv) The transfer of oxidative equivalents from Prx2 to STAT3 depends on the presence of AnxA2. (v) Prx2- and AnxA2-dependent STAT3 oxidation occurs in association with membranes. Combining these insights, we conclude that the Prx2-STAT3 redox relay is part of a highly organized membrane signaling microdomain.

## Results

### Peroxiredoxin-2 and STAT3 co-localize independently of and prior to H_2_O_2_-induced disulfide exchange

**Fig. 1:**
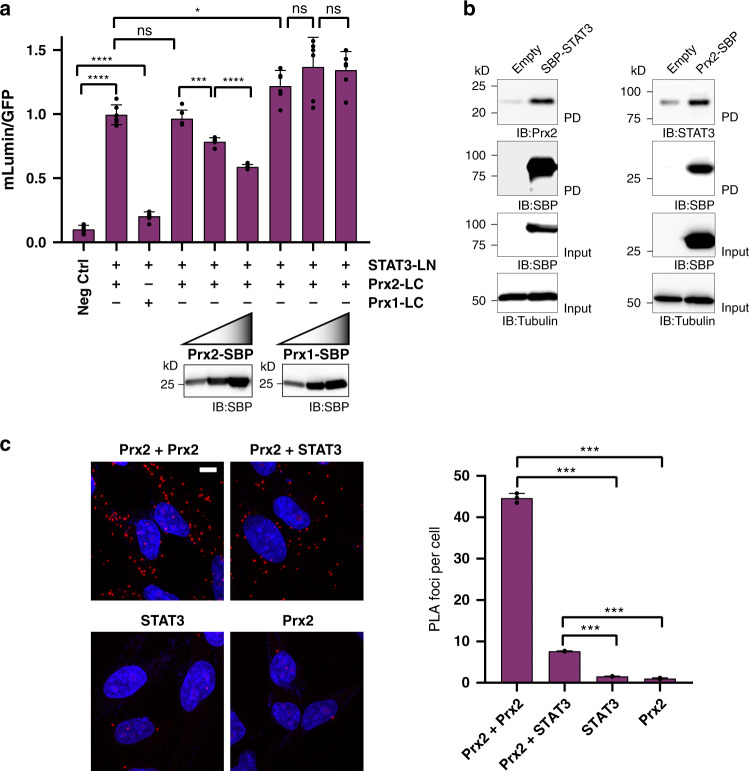
Peroxiredoxin 2 and STAT3 co-localize independently of and prior to H_2_O_2_-induced disulfide exchange. **a** Bimolecular fluorescence complementation: STAT3-LN interacts more strongly with Prx2-LC than with Prx1-LC (bars 2-3). Fluorescence complementation between STAT3-LN and Prx2-LC can be competed with Prx2-SBP (bars 4–6), but not with Prx1-SBP (bars 7–9). The negative control (bar 1) indicates non-specific complementation between unfused C- and N-terminal domains of mLumin (LC and LN, respectively). The immunoblots visualize the stepwise increase of Prx2-SBP and Prx1-SBP expression. Based on *n* = 6 independent experiments with *n* = 6 technical replicates each. IB: immunoblot. Source data are provided as a Source data file. **b** Co-precipitation: SBP-STAT3 co-precipitates Prx2 (left panels), and Prx2-SBP co-precipitates STAT3 (right panels). Blots are representative of *n* = 5 independent experiments. PD pulldown; IB immunoblot. Source data are provided as a Source data file. **c** Proximity ligation: Endogenous (untagged) Prx2 and STAT3 form a complex. Representative images (left panels) show PLA foci in red and DAPI in blue. The Prx2-Prx2 interaction (homo-oligomer formation) was used as a positive control, and single antibody experiments were included as negative controls. Scale bar: 10 µm. The bar graph (right panel) quantifies the number of PLA foci per cell (*n* = 50–60 cells in two technical replicates). The results are representative of *n* = 3 independent experiments. All bar charts in this figure represent the mean ± SD. *p < 0.05; ***p < 0.001; ****p < 0.0001; ns not significant; based on a two-tailed unpaired *t*-test.

We started by asking if STAT3 and Prx2 interact independently of and prior to pro-oxidative events that trigger disulfide exchange between the two proteins. To address this question, we first used a bimolecular fluorescence complementation (BiFC) approach. The N-terminal half of mLumin (LN) was fused to the C-terminus of STAT3, and the C-terminal half of mLumin (LC) to the C-terminus of either Prx2 or Prx1. HEK293 MSR cells stably expressing STAT3-LN were transiently transfected with either Prx2-LC or Prx1-LC (Supplementary Fig. 1a, left panel). Co-expression of Prx2-LC and STAT3-LN led to substantial fluorescence complementation. In contrast, co-expression of Prx1-LC and STAT3-LN yielded little fluorescence, only slightly higher than the negative control (Fig. 1a, bars 1–3, and Supplementary Fig. 1a, right panel). Moreover, complementation between STAT3-LN and Prx2-LC was competed by co-expression of Prx2, but not by co-expression of Prx1 (Fig. 1a, bars 4–6 and 7–9), indicating that STAT3 prefers to interact with Prx2 over Prx1. Microscopic visualization of mLumin complementation in cells expressing STAT3-LN and Prx2-LC revealed puncta, which were much less apparent in cells expressing STAT3-LN and Prx1-LC (Supplementary Fig. 1b), suggesting that the two proteins only form a close complex in specific, spatially confined locations. We then confirmed the interaction between Prx2 and STAT3 by Förster Resonance Energy Transfer (FRET), using Cerulean-STAT3 with either Prx2-Venus or Venus-Prx2 fusion proteins (Supplementary Fig. 1c). For further confirmation, we also performed co-precipitation experiments using the streptavidin binding peptide (SBP) tag: Ectopically expressed SBP-STAT3 co-precipitated endogenous Prx2 and ectopically expressed Prx2-SBP co-precipitated endogenous STAT3 (Fig. 1b). Since BiFC and FRET required tagging and ectopic expression of both interaction partners, and co-IP was performed by tagging one of the two interaction partners, we asked if the interaction of the endogenous (untagged) proteins can also be demonstrated. To this end, we used the proximity ligation assay (PLA). As a positive control, we used two different primary antibodies against Prx2 to demonstrate its well-established self-assembly into homo-oligomers (Fig. 1c, upper left panel). The in situ co-proximation of Prx2 and STAT3 was less prominent, but significantly above background (Fig. 1c, upper right panel). Taken together, these results confirmed that Prx2 and STAT3 interact non-covalently, in unstimulated cells, and prior to H_2_O_2_-induced disulfide exchange. STAT3 exhibits selectivity in its interaction with Prx2 over the closely related Prx1. Moreover, the interaction appears to be highly localized and restricted to small subpopulations of the overall Prx2 and STAT3 pools.

### The N-terminal domain of STAT3 is involved in the interaction with Prx2

**Fig. 2:**
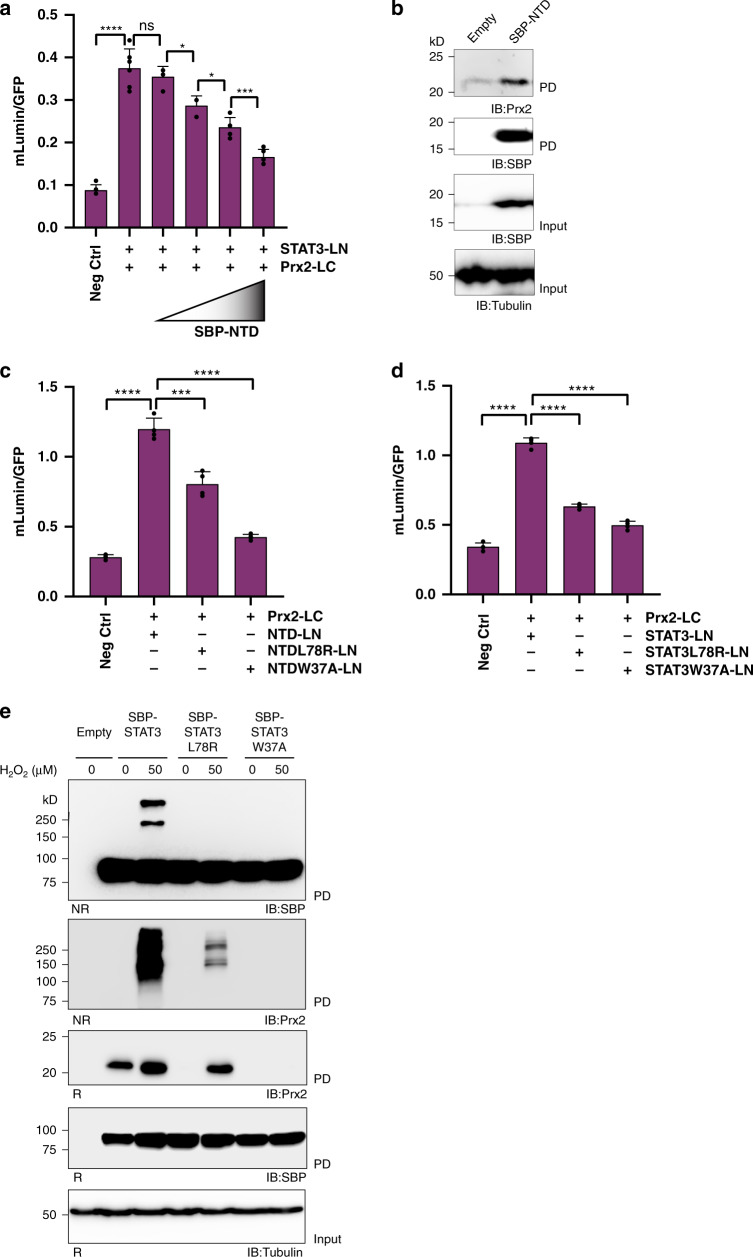
The N-terminal domain of STAT3 is involved in the interaction with Prx2. **a** The STAT3-Prx2 interaction can be competed with the NTD of STAT3. HEK293 MSR cells stably expressing Prx2-LC were co-transfected with STAT3-LN and increasing concentrations of SBP-tagged NTD. The bar charts in this figure show the mean ± SD from *n* = 6 independent experiments with *n* = 6 technical replicates each. **b** The NTD of STAT3 co-precipitates endogenous Prx2. The immunoblot is representative of *n* = 3 independent experiments. PD pulldown; IB immunoblot. Source data are provided as a Source data file. **c** Point mutations at the NTD surface disrupt the interaction between the NTD and Prx2. HEK293 MSR cells stably expressing Prx2-LC were transfected with NTD-LN, NTD(L78R)-LN or NTD(W37A)-LN. The corresponding immunoblot is shown in Supplementary Fig. 2a. The bar charts in this figure show the mean ± SD from *n* = 4 independent experiments with *n* = 6 technical replicates each. **d** Point mutations at the NTD surface disrupt the interaction between full-length STAT3 and Prx2. HEK293 MSR cells stably expressing Prx2-LC were transfected with STAT3-LN, STAT3(L78R)-LN or STAT3(W37A)-LN. The corresponding immunoblot is shown in Supplementary Fig. 2b. The bar charts in this figure show the mean ± SD from *n* = 4 independent experiments with *n* = 6 technical replicates each. **e** Point mutations at the NTD surface disrupt the formation of STAT3 oxidation products (top panel) and of Prx2-STAT3 disulfide exchange intermediates (second panel from top). The blots are representative of *n* = 3 independent experiments. PD pulldown; IB immunoblot. Source data are provided as a Source Data file. *P < 0.05; ***P < 0.001; ****P < 0.0001; ns not significant; based on a two-tailed unpaired *t*-test.

Next, we asked which domain of STAT3 may be involved in the interaction with Prx2. Since the N-terminal domain (NTD) has previously been shown to mediate both STAT3 oligomerization and interactions between STAT3 and other proteins^11^, we considered its involvement. We reasoned that if the NTD is involved in the interaction with Prx2, overexpression of a soluble NTD domain may compete the Prx2-STAT3 interaction. Indeed, increasing concentrations of ectopically expressed SBP-tagged NTD (SBP-NTD) competed the interaction between STAT3 and Prx2, as measured by BiFC (Fig. 2a). Furthermore, SBP-NTD co-precipitated endogenous Prx2 (Fig. 2b). Likewise, BiFC demonstrated an interaction between NTD-LN and Prx2-LC (Fig. 2c, bars 1–2). The NTD is known to have two interaction surfaces. We introduced one mutation (L78R) known to interrupt the dimerization interface^12^ and another one (W37A) known to interrupt the tetramerization interface^13^. The L78R mutation diminished the interaction between NTD-LN and Prx2-LC and the W37A mutation almost abolished it (Fig. 2c, bars 3–4, and Supplementary Fig. 2a). The same mutations had a very similar effect in the context of full-length STAT3 (Fig. 2d and Supplementary Fig. 2b). Therefore, we asked if the mutations would also diminish or abolish the transfer of oxidizing equivalents from Prx2 to STAT3. Indeed, both mutants did not form detectable amounts of oxidation products (Fig. 2e, top panel). Likewise, the formation of Prx2-STAT3 mixed disulfide intermediates was either greatly diminished (L78R) or absent (W37A) (Fig. 2e, second panel from top). In conclusion, the interaction between Prx2 and STAT3 involves the NTD of STAT3, as point mutations in the NTD disrupt both non-covalent and covalent interactions with Prx2 and consequently prevent STAT3 oxidation.

### Annexin A2 interacts with both Prx2 and STAT3

**Fig. 3:**
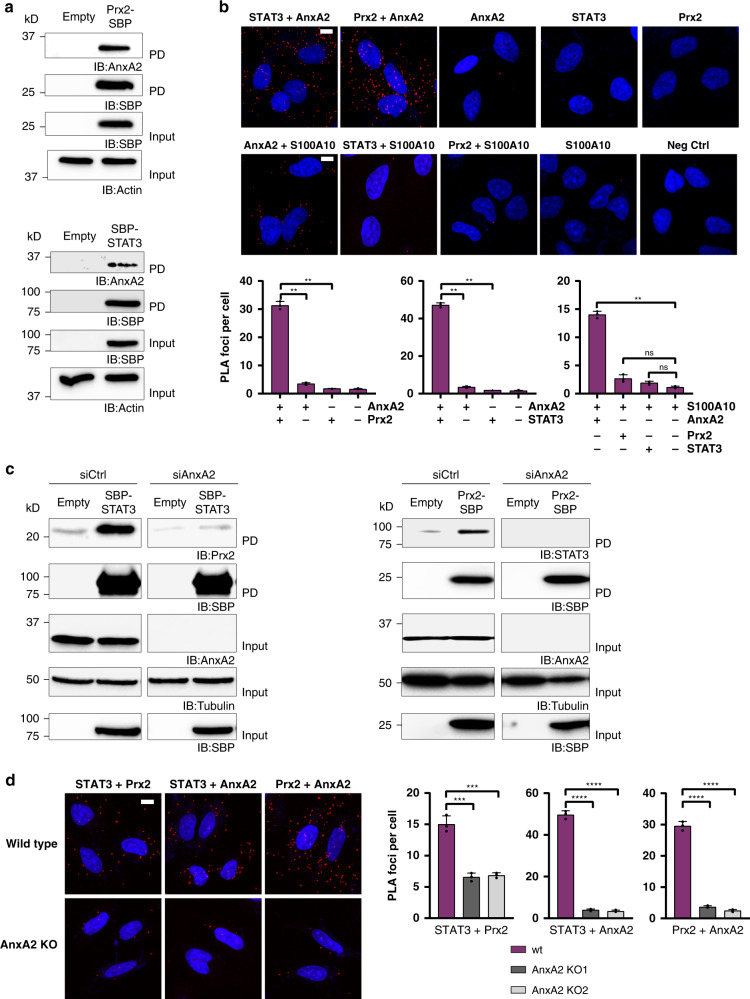
Annexin A2 interacts with both Prx2 and STAT3. **a** Co-precipitation: Both Prx2 (upper panels) and STAT3 (lower panels) co-precipitate AnxA2. The blots are representative of *n* = 3 independent experiments. PD pulldown; IB immunoblot. Source data are provided as a Source data file. **b** Proximity ligation: AnxA2 interacts with Prx2 and STAT3 (upper panels). S100A10 interacts with AnxA2 (positive control), but not with Prx2 or STAT3 (middle panels). Single antibody experiments are included as negative controls. Representative images show PLA foci in red and DAPI in blue. Scale bars: 10 µm. The bar graphs (lower panels) quantify the number of PLA foci per cell (*n* = 50–60 cells in *n* = 2 technical replicates). The results are representative of *n* = 3 independent experiments. **c** Depletion of AnxA2 by siRNA diminishes the association between Prx2 and STAT3. Co-precipitation of Prx2 by SBP-STAT3 (left panels) and co-precipitation of STAT3 by Prx2-SBP (right panels). The blots are representative of *n* = 3 independent experiments. PD pulldown; IB immunoblot. Source data are provided as a Source data file. **d** Deletion of AnxA2 compromises the Prx2-STAT3 interaction as detected by PLA. Representative images show PLA foci in red and DAPI in blue (left panels). Scale bar: 10 µm. The bar graphs (right panels) show the frequency of PLA foci in AnxA2 KO cells relative to wild type cells (*n* = 40–50 cells in *n* = 3 technical replicates), using two independent clones of AnxA2 KO cells. The interaction of AnxA2 with Prx2 or STAT3 in AnxA2 KO cells represents the background signal. The results are representative of *n* = 3 independent experiments. All bar charts in this figure represent the mean ± SD. **p < 0.01; ***p < 0.001; ****p < 0.0001; ns not significant; based on a two-tailed unpaired *t*-test.

The above results indicated that subpopulations of STAT3 and Prx2 interact in a selective manner (Prx1 vs. Prx2), dependent on distinct structural elements (NTD), to form part of the same complex (as revealed by BiFC, FRET, co-IP and PLA). We then wondered if the interaction is direct or dependent on the presence of another protein to scaffold the interaction. Our attempts to demonstrate a direct interaction of the recombinant proteins in vitro were not successful. We therefore asked if there are proteins known to interact with both Prx2 and STAT3. Inspection of the BioGRID protein interaction repository revealed a single common interaction partner, annexin A2 (AnxA2): Interactions between Prx2 and AnxA2 and between STAT3 and AnxA2, respectively, have been reported independently of each other^9,14^. In addition, our lab previously identified AnxA2 as an interaction partner of Prx2^9^. Thus, we considered the possibility that AnxA2 acts as a scaffold for the Prx2-STAT3 interaction. First, we aimed to confirm that the three proteins interact with each other inside the cell. Prx2-SBP and SBP-STAT3 independently co-precipitated endogenous AnxA2 (Fig. 3a) and endogenous AnxA2 co-immunoprecipitated endogenous STAT3 (Supplementary Fig. 3a). PLA further verified interactions between endogenous untagged proteins (Fig. 3b): As a positive control, AnxA2 was confirmed to interact with S100A10, a well-established interaction partner. Prx2 and STAT3 were both found to interact with AnxA2, but not with S100A10, indicating that S100A10 is not part of the Prx2-STAT3-AnxA2 complex. Of note, the interaction between STAT3 and AnxA2 is neither affected by Prx2 depletion (Supplementary Fig. 3b), nor by the NTD mutations (Supplementary Fig. 3c).

We then asked if AnxA2 is actually required for the formation of the Prx2-STAT3 complex. To this end, we depleted AnxA2 by siRNA and then performed co-precipitation experiments. Upon depletion of AnxA2, co-precipitation of endogenous Prx2 by SBP-STAT3 (Fig. 3c, left panels) and co-precipitation of endogenous STAT3 by Prx2-SBP (Fig. 3c, right panels) was strongly diminished. Upon deletion of AnxA2 (Supplementary Fig. 3d), the PLA signal for the Prx2-STAT3 interaction was reduced (Fig. 3d). Taken together, these results suggested that AnxA2 interacts with both Prx2 and STAT3 and is required for bringing STAT3 and Prx2 together.

### STAT3 oxidation depends on AnxA2

**Fig. 4:**
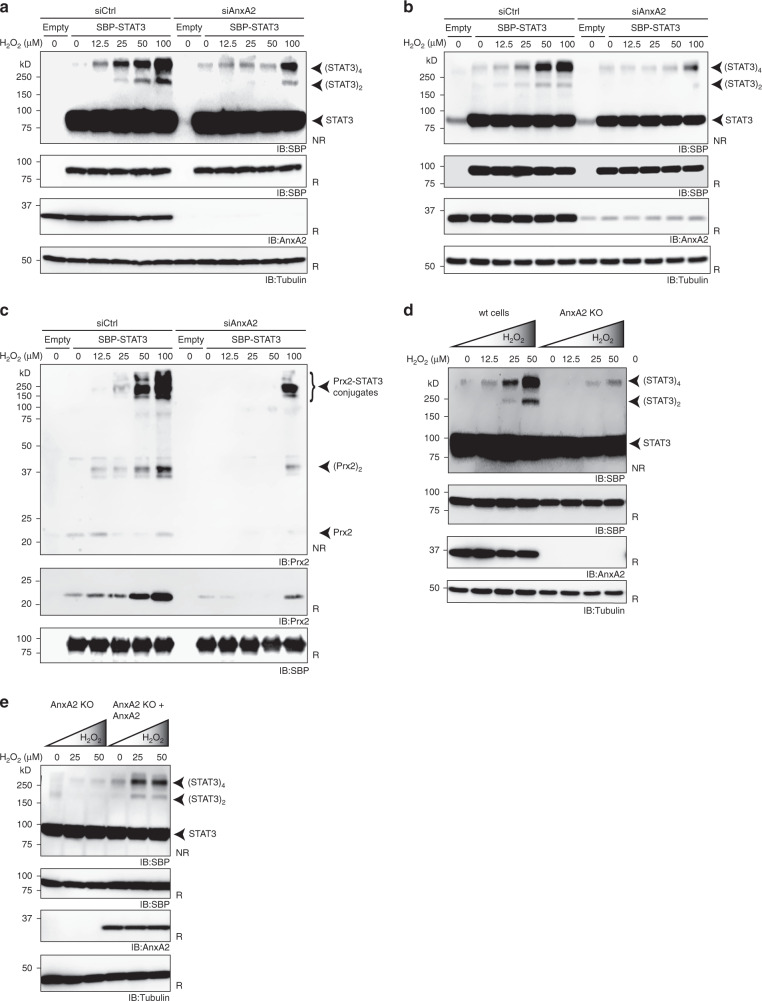
STAT3 oxidation depends on AnxA2. **a** H_2_O_2_-induced STAT3 oxidation is diminished upon depletion of AnxA2 in HEK293 MSR cells. The formation of covalent (disulfide-linked) STAT3 dimers and tetramers in response to increasing concentrations of exogenously applied H_2_O_2_ is visualized by immunoblotting on non-reducing SDS-PAGE. **b** The same experiment as in **a**, performed in U2OS cells. **c** The formation of Prx2-STAT3 disulfide exchange intermediates, representing the transfer of oxidizing equivalents from Prx2 to STAT3, is largely abolished upon depletion of AnxA2 in HEK293 MSR cells. **d** H_2_O_2_-induced STAT3 oxidation is largely abolished upon deletion of AnxA2 (AnxA2 KO) in HEK293 MSR cells. **e** Re-expression of ectopic AnxA2 in AnxA2 KO cells recovers H_2_O_2_-induced STAT3 oxidation. All immunoblots shown in this figure are representative of *n* = 3 independent experiments. IB immunoblot; NR/R Non-reducing/Reducing gel electrophoresis. All source data for this figure are provided as a Source data file.

The finding that AnxA2 is important for the co-proximation of Prx2 and STAT3 raised the question if AnxA2 is also required for the transmission of oxidative equivalents from Prx2 to STAT3. To address this question, we compared AnxA2-proficient and -deficient cells expressing SBP-STAT3. As previously docmented^6^, STAT3 forms oxidation products (covalent dimers and tetramers) in response to H_2_O_2_. However, upon depletion of AnxA2, STAT3 oxidation was considerably diminished in two independent cell lines (Fig. 4a, b). Likewise, Prx2-STAT3 mixed disulfide intermediates, formed during the transfer of oxidizing equivalents, were strongly diminished in AnxA2-depleted cells relative to AnxA2-proficient cells (Fig. 4c). Moreover, STAT3 oxidation was strongly diminished in AnxA2 KO cells (Fig. 4d, Supplementary Fig. 4a) and the ectopic re-expression of AnxA2 in AnxA2 KO cells largely rescued the formation of STAT3 oxidation products (Fig. 4e, Supplementary Fig. 4b, c). AnxA2 has been reported to harbor two redox-sensitive cysteines, Cys-8 and Cys-132^15^. We therefore asked if these cysteines are needed for Prx2-mediated STAT3 oxidation. We expressed corresponding AnxA2 cysteine mutants (C8S and C132S) in AnxA2 KO cells and found them to rescue the oxidation of STAT3, albeit somewhat less efficiently than wild type AnxA2 (Supplementary Fig. 4d). Taken together, these results show that Prx2-mediated oxidation of STAT3 depends on the presence of AnxA2, but not on the presence of AnxA2 cysteines.

### STAT3 oxidation occurs at the membrane and dampens transcriptional cytokine responses

**Fig. 5:**
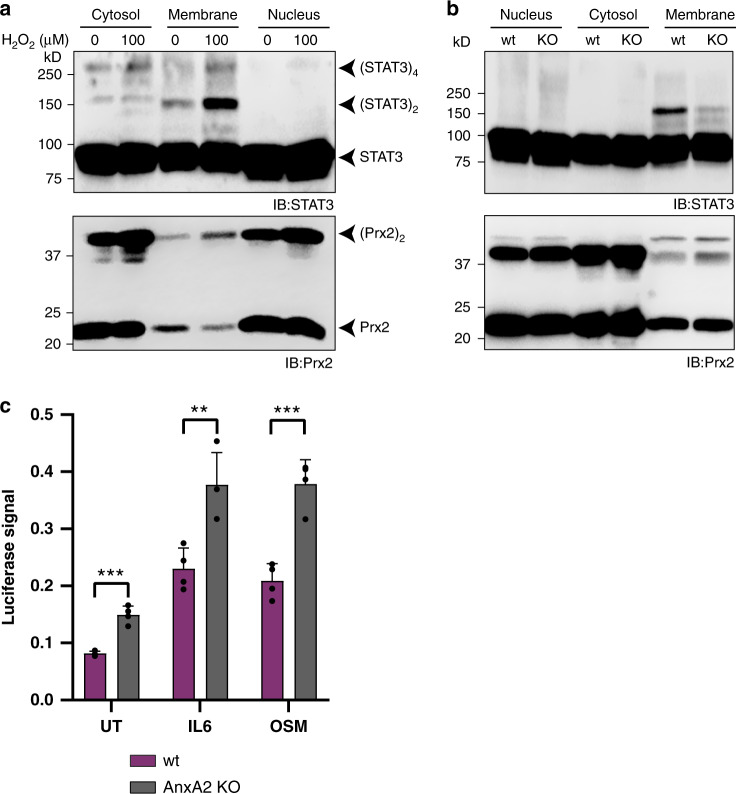
AnxA2-dependent STAT3 oxidation occurs at the membrane and affects the transcriptional response. **a** STAT3 oxidation is associated with membranes. HEK293 MSR cells were pulsed with 100 µM H_2_O_2_ for 2 min and then fractionated into cytosol, membranes, and nuclei. The corresponding reducing immunoblot is shown in Supplementary Fig. 5a. The blots are representative of *n* = 3 independent experiments. IB immunoblot. Source data are provided as a Source data file. **b** Membrane-associated STAT3 oxidation depends on AnxA2. HEK293 MSR wild type and AnxA2 KO cells were fractionated into cytosol, membranes and nuclei. The corresponding reducing immunoblot is shown in Supplementary Fig. 5b. The blots are representative of *n* = 3 independent experiments. IB immunoblot. Source data are provided as a Source data file. **c** AnxA2 deficiency increases STAT3-dependent transcription from the serum inducible element (SIE) promotor. STAT3 signaling was induced by either IL-6 or OSM treatment and SIE promotor activity was measured with a luciferase reporter. Bars represent the mean (±SD) of *n* = 3 biological replicates. **p < 0.01; ***p < 0.001; based on a two-tailed unpaired *t*-test.

AnxA2 has been described to act as a membrane scaffold protein recruiting various client proteins to membrane signaling domains^16^. We therefore asked if Prx2- and AnxA2-dependent STAT3 oxidation takes place in association with membranes. This was already suggested by our previous finding that IL-6 triggers the oxidation of STAT3^6^, implicating NADPH oxidase (NOX) activity coupled to cytokine receptor stimulation. To address this question, we fractionated cells. We found oxidized STAT3 to reside almost exclusively in the membrane fraction (Fig. 5a, upper panel, and Supplementary Fig. 5a). A small population of oxidized Prx2 was also associated with the membrane fraction, as expected (Fig. 5a, lower panel, and Supplementary Fig. 5a). Importantly, the membrane fraction derived from AnxA2 KO cells was almost devoid of oxidized STAT3 (Fig. 5b, and Supplementary Fig. 5b), in agreement with the notion that AnxA2 facilitates membrane-associated STAT3 oxidation.

We previously observed that IL-6- or oncostatin-M (OSM)-induced STAT3 oxidation dampens transcription from the serum inducible element (SIE)^6^ and that depletion of Prx2 (which prevents STAT3 oxidation) increases SIE transcription. These findings predicted that depletion of AnxA2 (which also prevents STAT3 oxidation) should show the same effect. Indeed, deletion of AnxA2 enhanced IL-6- or OSM-induced transcription from the SIE promoter (Fig. 5c). In line with the finding that STAT3 oxidation decreases its recruitment to the c-fos promoter^17^, we also observed an increase in IL6-induced FOS expression upon depletion or deletion of AnxA2 (Supplementary Fig. 5c, d). However, the depletion or deletion of AnxA2 had no effect on IL6-induced Tyr-705 phosphorylation of STAT3 (Supplementary Fig. 5e, f). Taken together, these results support the notion that AnxA2-dependent and Prx2-mediated oxidation of STAT3 occurs at membranes (plasma and/or endomembranes) and affects cytokine-induced downstream signaling.

## Discussion

In this study, we investigated how a peroxiredoxin and a redox-regulated target protein are brought into proximity to form a functional redox relay. To this end, we focused on the specific case of the Prx2-STAT3 redox relay.

The first question we asked was whether the two relay partners come together prior to any oxidative event, while they are still in the reduced state. We show that Prx2 and STAT3 interact with each other non-covalently, in the absence of pro-oxidative stimuli, and before disulfide exchange intermediates or oxidation products can be detected. The interaction is restricted to very small subpopulations of both proteins, suggesting a specific microenvironment. Thus, it seems that Prx2 reacts with H_2_O_2_ while being part of a pre-assembled complex, and therefore is poised to transfer its oxidative state to the proximal client. It makes sense that target thiols should be pre-positioned to “capture” the sulfenic acid (SOH) as soon as it becomes available on the Prx2 surface, considering that the SOH is prone to react with the resolving cysteine (or glutathione^18^) and therefore has a limited half-life.

We then asked if there is evidence for the selectivity of the pairing. On the one hand, we found that STAT3 strongly prefers Prx2 over Prx1, although these two Prxs are very similar in structure and function. However, these two isoforms accumulate the SOH at different threshold concentrations of H_2_O_2_, presumably to allow for step-wise sensing of H_2_O_2_^19^. Prx2 is most sensitively oxidized and will accumulate the SOH already at low nanomolar H_2_O_2_ concentrations. This suggests that Prx2 is the preferred sensor for the smallest changes in local H_2_O_2_ availability, as would be needed for (non-stress) signal transduction. Prx1 is a less sensitive sensor, accumulating the SOH only at higher H_2_O_2_ concentrations^19^, potentially reflecting stress conditions. Thus, it may not be a coincidence that Prx2 has been found in a relay with a transcription factor^6^ and Prx1 in a relay with a stress signaling kinase^7^.

On the other hand, a single point mutation in the NTD of full-length STAT3 completely abolished both non-covalent and covalent interactions with Prx2, and, consequently, the formation of STAT3 oxidation products. The mutation is known to alter the STAT3 oligomerization interface which is also the interface for interactions with other proteins^11^. Thus, Prx2 discriminates between structural variants of STAT3,  while STAT3 discriminates between the closely related Prx1 and Prx2 isoforms. Hence, the interaction appears to be selective in both directions. The pre-assembly prior to oxidative transfer, together with the mutual selectivity, strongly speak against the “null hypothesis”, i.e. that Prxs, once oxidized, indiscriminately transfer oxidizing equivalents to other proteins by random diffusion and chance encounters.

The next question we asked is whether Prx2 is guided towards STAT3 by virtue of a direct protein–protein-interaction. We failed to reconstitute a Prx2-STAT3 relay from pure proteins in vitro, and the recently attempted in vitro reconstitution of another redox relay (Prx2-CRMP2) also failed^8^. These negative results can be taken to suggest that one (or more) additional proteins are needed to scaffold the interaction. This possibility is obvious because the first redox relay that was discovered, Orp1-Yap1, does not work without a dedicated scaffold protein, Ybp1^20,21^. There are also physicochemical arguments, supported by mathematical models, suggesting that a Prx-based redox relay can only work when it is templated^22,23^. In this study, we show that the Prx2-STAT3 redox relay indeed requires (at least) one other protein, namely AnxA2. Like other annexins, AnxA2 has been described as a membrane scaffold protein, forming networks on membrane surfaces and providing a recruitment platform for other proteins, to organize membrane domains^24^. Our findings reveal that AnxA2 enables proximity between Prx2 and STAT3, the transfer of oxidizing equivalents from Prx2 to STAT3, and the formation of STAT3 oxidation products. Accordingly, the deletion or depletion of AnxA2 strongly diminished (by 85–95%) the non-covalent and covalent interactions between Prx2 and STAT3, and the formation of covalent STAT3 oligomers (as monitored by co-IP and gel mobility). In contrast, the effect of the AnxA2 deletion on the formation of STAT3-Prx2 PLA foci was less pronounced (≈60% reduction). However, given that the classical PLA method (using oligo-coupled secondary antibodies) can bridge proteins over a distance of up to 80 nm^25^, we suspect that Prx2 and STAT3 remain in relative proximity within membrane patches, even though their direct interaction is disrupted in the absence of AnxA2.

Interestingly, the NTD point mutations that diminished or abolished the interaction of STAT3 with Prx2 did not affect its interaction with AnxA2. Thus, while the STAT3-Prx2 association requires both AnxA2 and the NTD, the STAT3-AnxA2 association does not require the NTD, nor the presence of Prx2. This finding can be interpreted in several ways. For example, it is conceivable that the binding of AnxA2 to STAT3 occurs outside the NTD and changes the conformation of STAT3 to allow interaction between the NTD and Prx2. It is also conceivable that AnxA2 binds STAT3 independently of its oligomeric state, while Prx2 can only bind dimeric and/or tetrameric STAT3 (the formation of which is prevented by the NTD mutation). Since all three interaction partners are oligomeric proteins (and known to switch between alternative oligomeric states) it seems possible that their relative oligomeric stoichiometry plays a role in the assembly of the complex.

A more detailed understanding of the structural organization will require the in vitro reconstitution of a functional AnxA2-Prx2-STAT3 redox relay complex from recombinant proteins. Unfortunately, given the fact that AnxA2 assembles on membrane surfaces and is subject to various posttranslational modifications, reconstitution is likely to require conditions that are not easily mimicked in vitro. Moreover, reconstitution may also require additional protein components. We consider the in vitro reconstitution of a membrane-associated redox relay complex a long-term effort beyond the scope of the present study.

It is interesting to note that AnxA2 has been proposed to be a redox-regulated protein. It possesses four cysteine residues, two of which were reported to be redox-sensitive^15,26^. S-glutathionylation of AnnexinA2 has been linked to its phospholipid and F-actin binding activity^15^. Moreover, we recently identified AnxA2 as a disulfide exchange partner of Prx2^9^, suggesting that Prx2 can transfer oxidation to AnxA2, probably in a way similar to STAT3 oxidation. However, mutagenesis of AnxA2 cysteines affected STAT3 oxidation only weakly and in none of our experiments did we detect disulfide exchange intermediates involving AnxA2. Nevertheless, a role for the AnxA2 cysteines in the context of STAT3 redox regulation cannot be excluded and deserves further investigation.

Only small sub-populations of STAT3 and Prx2 assemble into the redox relay complex. A very rough estimate is that at most 1-5% (but probably less) of total STAT3 becomes oxidized under conditions of H_2_O_2_ exposure, and the proportion is even lower when STAT3 oxidation is induced by cytokine treatment^6^. Considering protein copy number estimates determined for HeLa cells^27^, Prx2 and AnxA2 are highly expressed proteins (with similar copy numbers), both being more than 3 orders of magnitude more abundant than STAT3. From this, it would seem that STAT3 should be the limiting factor for complex assembly, yet only a small fraction of total STAT3 is in the complex and oxidizable. Formation of the complex may actually be limited by specific posttranslational modifications in any of the components. Also, we cannot exclude the possibility that complex formation is limited by another (currently unknown) protein.

The functional role of STAT3 oxidation in the context of canonical STAT3 signaling and/or other STAT3 functions remains incompletely understood. We observed STAT3 oxidation to occur in association with membranes, in line with STAT3 oxidation being triggered by cytokine receptor stimulation^6^. STAT3 oxidation does not seem to affect STAT3 tyrosine phosphorylation, yet it negatively influences downstream transcriptional activity. It may be speculated that STAT3 oxidation serves as a mechanism that limits the strength and/or duration of STAT3 signaling in response to changing metabolic conditions. We have reported earlier that the momentary STAT3 redox state is determined by the balance between Prx2-dependent oxidation and thioredoxin (Trx)-dependent reduction^6^. Thus, the balance between STAT3 oxidation and reduction within membrane domains likely makes STAT3 signaling responsive to oxygen availability (which determines H_2_O_2_ production) and the intracellular NADPH/NADP^+^ ratio (which determines Trx-mediated reduction). For example, EGF receptor signaling has been found to respond to oxygen gradients through the conversion of O_2_ to H_2_O_2_ and local protein redox regulation^28^. Most recently, Busker et al. described inhibitors of the thioredoxin system that inhibit STAT3 signaling in cancer cells with nanomolar potency^29^. They demonstrate that STAT3 signaling is blocked when Prx2-dependent STAT3 oxidation is not counterbalanced by the Trx/TrxR/NADPH system, leading to the death of tumor cells that depend on STAT3 signaling. We expect that further mechanistic understanding of the STAT3 redox relay complex will allow to dissect and manipulate the physiological roles of STAT3 oxidation in future studies.

## Methods

### Cell lines, antibodies, and chemicals

HEK293 MSR (GripTite™, Thermo Fisher Scientific), HeLa (ATCC), U2OS (ATCC) and Phoenix-AMPHO cells (ATCC) were maintained in Dulbecco’s Modified Eagle Medium (DMEM; Life Technologies), supplemented with 10% (v/v) bovine calf serum (Life technologies), 2 mM l-glutamine (Life Technologies) and 50 units/ml penicillin and streptomycin (Life Technologies). The medium for HEK293 MSR cells additionally contained 50 units/ml Geneticin (Life Technologies). All cell lines were confirmed to be free of mycoplasma, viral infections and contaminations with other cell lines, based on multiplex PCR and SNP profiling. Antibodies used in this study were mouse anti-SBP (Santa Cruz, sc101595), rabbit anti-tubulin (Cell Signaling, 2128), mouse anti-actin (Sigma, A5441), rabbit anti-HA tag (Cell Signaling, 3724), rabbit anti-MYC tag (Cell Signaling, 2278), rabbit anti-STAT3 (Cell Signaling, 12640), mouse anti-STAT3 (Cell Signaling, 9139), rabbit anti-Prx2 (abcam, 109367), mouse anti-Prx2 (Thermo Scientific, LF-MA0144), mouse anti-Annexin A2 (Santa Cruz, sc47696), rabbit anti-Annexin A2 (Cell Signaling, 8235), mouse anti-S100A10 (Cell Signaling, 5529), goat anti-lamin B (Santa Cruz, sc6217), mouse anti-Na^+^/K^+^ ATPse (Santa Cruz, sc48345) and mouse anti-STAT3 phospho (Tyr705) (Cell Signaling, 9138). All antibodies were used at a dilution of 1:1,000. The secondary antibodies used for western blotting in this study were anti-goat (Santa Cruz, sc2020), anti-mouse (Jackson immunoResearch, 115-035-146) and anti.rabbit (Jackson ImmunoResearch, 111-035-144). All secondary antibodies were used at a dilution of 1:10,000. All chemicals were purchased from Sigma-Aldrich, unless stated otherwise.

### DNA constructs

The gateway destination vectors pDest HA-LN151 and pDest MYC-LC151, described previously^30^, were kindly provided by Stephan Pusch (German Cancer Research Center). The ORFs for peroxiredoxin 1 and 2 and STAT3 were provided by the DKFZ Genomics & Proteomics Core Facility. The Cerulean-5-Venus FRET control construct was obtained from Addgene (#31182). All expression plasmids were constructed by either using the Gateway™ recombination system (Life Technologies) or the Gibson Assembly Cloning Kit (New England Biolabs). Primers for Gibson Assembly were designed using the NEBuilder Assembly Tool. Point mutations were introduced with the QuikChange™ site-directed mutagenesis kit (Agilent). Primers used for mutagenesis are listed in Supplementary Table 1. Expression plasmids used in this study were pcDNA3.1(-) STAT3-HA-LN151, pLPCX STAT3-HA-LN151, pcDNA3.1(-) Prx2-MYC-LC151, pLPCX Prx2-MYC-LC151, pcDNA3.1(-) Prx1-MYC-LC151, pcDNA3.1(-) MYC-LC151, pcDNA3.1(-) HA-LN151, pcDNA3.1(-) NTD-HA-LN151, pcDNA3.1(-) Cerulean-STAT3, pcDNA3.1(-) Prx2-Venus, pcDNA3.1(-) Venus-Prx2, pcDNA3.1(-) Cerulean-5-Venus, pcDNA3.1(-) Cerulean-P2A-Venus, pcDNA3.1(-) Prx2-SBP, pcDNA3.1(-) SBP-STAT3, pcDNA3.1(-) SBP-NTD and pcDNA3.1(-) Annexin A2, and pGL4.47(luc2P/SIE/Hygro) (Promega).

### Transfection and stable cell line generation

HEK293 MSR cells were transfected using polyethylenimine (Polysciences) as described previously^31^. U2OS cells were transfected using Lipofectamine 3000 following the manufacturer’s instructions. For experiments performed in 96-well plates, HEK293 MSR cells were transfected using Lipofectamine 2000 following the manufacturer’s instructions. For generation of stable cell lines, pLPCX retroviral expression vectors were transfected into the packaging cell line Phoenix-AMPHO. After 24 h, viral supernatant was collected, filtered through a 0.45 μm cellulose acetate filter and then used to infect freshly thawed target cells. Transduced cells were selected with puromycin, expanded, and frozen for later use.

### Bimolecular fluorescence complementation (BiFC)

In all, 4 × 10^4^ HEK293 MSR cells stably expressing either STAT3-HA-LN or Prx2-MYC-LC were seeded into wells of a 96-well plate. The next day, cells were transfected with a plasmid encoding LN- or LC-tagged proteins of interest (Prx2, Prx1, STAT3 or NTD.) A plasmid encoding GFP was co-transfected for normalization purposes. For the Prx2/Prx1 competition experiment (Fig. 1a), cells stably expressing STAT3-HA-LN were transfected with plasmids encoding Prx2-MYC-LC and Prx1/2-SBP in defined molar ratios (0.5, 1, and 2). For the NTD competition experiment (Fig. 2a), cells stably expressing Prx2-MYC-LC were transfected with plasmids encoding STAT3-HA-LN and SBP-NTD in defined molar ratios (0.5, 1, 2, 4). All transfections were performed using Lipofectamine 2000 as per manufacturer’s instructions. 48 h after transfection, medium was replaced by pre-warmed Dulbecco’s Phosphate Buffered Saline (DPBS). mLumin fluorescence (Ex 560 nm/Em 630 nm) and GFP fluorescence (Ex 480 nm/Em 520 nm) were recorded with a microplate reader (BMG ClarioStar™). mLumin fluorescence intensity was normalized to GFP fluorescence intensity. For microscopy, HEK293 MSR cells co-expressing STAT3-HA-LN and Prx2-Myc-LC, STAT3-HA-LN and Prx1-Myc-LC or HA-LN and Myc-LC were fixed with 4% formaldehyde for 15 min, 48 h after transfection. After washing three times with DPBS, fixed cells were mounted with mounting medium containing DAPI. The cells were subsequently imaged with a confocal microscope (Zeiss LSM 710 ConfoCor 3). Samples were excited with the 561 nm laser line and fluorescence detected with a 600–680 nm filter. The objective was an EC Plan-Neofluar 40×/1.30 Oil DIC M27. Acquired images were processed in ImageJ.

### Proximity ligation assay

The proximity ligation assay (Duolink™, Sigma-Aldrich) was performed according to the manufacturer’s instructions. Briefly, HeLa cells were grown on imaging dishes (Ibidi), then fixed with ice-cold methanol for 7 min on ice. Cells were incubated with 3% BSA in DPBS for 1 h and then with primary antibodies overnight at 4 °C. Subsequently, cells were incubated with oligonucleotide-labeled secondary antibodies, followed by ligation, DNA amplification, and probe hybridization. Resulting signals were visualized by confocal microscopy.

### Co-precipitation by streptavidin affinity enrichment

Cells were transfected with SBP-STAT3, Prx2-SBP, SBP-NTD or empty vector in 15 cm^2^ dishes. After 48 h, cells were either pulsed with different concentrations of H_2_O_2_ for 2 min or left untreated (Figs. 2e and 4a–e). Cells were left untreated 48 h post transfection in Figs. 1b, 2b, 3a, c. Free thiols were then blocked with ice-cold 100 mM N-ethylmaleimide (NEM) in DPBS for 7 min. Cells were lysed with 1% Triton X-100 in TBS (50 mM Tris, 150 mM NaCl, pH 7.4) supplemented with protease inhibitor (cOmplete™, Roche). Post-nuclear lysates were incubated with 30 µl streptavidin-coupled sepharose beads (High Performance Streptavidin Sepharose, GE Healthcare) for 4 h. Beads were washed twice with 1% Triton X-100 and once with 0.1% Triton X-100 in TBS (Figs. 1b, 2b, 2e, and 3a, c). Beads were washed with 1% Triton X-100, 0.5 mM NaCl, 1 M urea in TBS, then with 1% Triton X-100 in TBS and finally with 0.1% Triton X-100 in TBS (Fig. 4a–e). Elution was performed with 60 µL 4 mM Biotin in TBS. The protein samples were subjected to non-reducing or reducing SDS-PAGE and immunoblot analysis.

### Immunoblot analysis

Protein samples were dissolved in SDS-PAGE sample buffer, with (reducing) or without 25 mM DTT (non-reducing). Samples were separated by SDS-PAGE and proteins transferred to polyvinyl difluoride (PVDF) membranes (Immobilon-P, Millipore) using a transfer tank (TE22, Hoefer). Membranes were probed with appropriate antibodies and chemiluminescent substrate (SuperSignal West Femto, Thermo Scientific).

### Depletion of AnxA2

Annexin A2 was depleted in HEK293 MSR or U2OS cells using ON-TARGET plus siRNA SMARTpool (Dharmacon) or a single pre-designed siRNA (Ambion) with the following sequence: GGAUAUUGCCUUCGCCUACTT (AM16708). The siRNAs were transfected using DharmaFECT 1 (Dharmacon) reagent as per manufacturer’s instructions. The ON-TARGET plus non-targeting pool (Dharmacon D-001810-10-05) or the Silencer™ negative control No. 1 siRNA (Ambion) were used as controls.

### Generation of AnxA2 knockout cells

Guide RNAs (gRNAs) targeting the AnxA2 gene were designed using the Alt-R™ CRISPR-Cas9 guide RNA tool (Integrated DNA technologies). The gRNAs used in this study were: CTCAGCATCAAAGTTAGTAT (ANXA2.1.AA) and AACTGATTGACCAAGATGCT (ANXA2.1.AC). The Cas9 ribonucleoprotein complex, containing the crRNA:tracrRNA duplex and the Cas9 nuclease protein (Integrated DNA technologies), was electroporated into HEK293 MSR or HeLa cells. Briefly, 1 × 10^5^ HEK293 MSR or HeLa cells were suspended in 9 μl Neon electroporation buffer R. The crRNA:tracrRNA duplex (1.8 μM final concentration), HiFi™ Cas9 nuclease (1.5 μM final concentration) and Cas9 electroporation enhancer (1.8 μM final concentration) were added to the cells. Electroporation settings were 1005 V, 35 ms, and 2 pulses for HeLa cells and 1600 V, 20 ms, and 1 pulse for HEK293 MSR cells. Post electroporation, cells were kept in a tissue culture incubator (37 °C, 5% CO_2_) for 72 h. Subsequently, single-cell clones were grown in a 96-well plate. Clones were expanded and Annexin A2 knockout clones were identified by immunoblotting.

### Membrane fractionation

In all, 7 × 10^6^ HEK293 MSR cells were seeded into 15 cm^2^ cell culture dishes and grown overnight. The next day, cells were treated with 100 µM H_2_O_2_ for 2 min or left untreated. Following treatment, the medium was aspirated, and cells were incubated for 7 min with ice-cold DPBS containing 100 mM NEM. Cells were harvested after washing them once with DPBS. Cytosolic, nuclear and plasma membrane fractions were prepared with the Minute™ Plasma Membrane Protein Isolation and Cell Fractionation Kit (Invent Biotechnologies) in accordance with the manufacturer’s instructions.

### Luciferase reporter assay

HEK293 MSR cells were seeded into 96-well plates and transfected with Lipofectamine 2000 in accordance with the manufacturer’s instructions. In all, 24 h post-transfection, cells were treated for 8 h with either 50 ng/ml Oncostatin M (Biomol, 97241) or 50 ng/mL IL-6/IL-6 receptor (R&D Systems, 206-IL and 227-SR). The luciferase assay (Dual-Glo™, Promega) was conducted according to the manufacturer’s protocol. Luminescence was measured with a microplate reader (Pherastar, BMG). The firefly luciferase signal was normalized to the *Renilla* luciferase signal.

### Reporting summary

Further information on research design is available in the Nature Research Reporting Summary linked to this article.

## Supplementary information

Supplementary Information

Reporting Summary

## Data Availability

All data generated or analyzed during this study are included in this published article and its supplementary information files; and are available from the corresponding author upon reasonable request. The BioGRID database (https://thebiogrid.org/) was used to identify Annexin A2 as a common interaction partner of Prx2 and STAT3. Source data are provided with this paper.

## Source data

Source Data
