# Supplementary material for: A role for annexin A2 in scaffolding the peroxiredoxin 2–STAT3 redox relay complex
Source: Nat Commun. 2020 Sep 9;11:4512. doi: 10.1038/s41467-020-18324-9 (PMC7481202; doi:10.1038/s41467-020-18324-9)
Supplement: Supplementary file 1 — Supplementary Information [file 41467_2020_18324_MOESM1_ESM.pdf]

Supplementary Information

A role for annexin A2 in organizing the peroxiredoxin 2 - STAT3 redox relay complex

Deepti Talwar[1,2], Joris Messens[3-5], Tobias P. Dick[1]

[1]Division of Redox Regulation, DKFZ-ZMBH Alliance, German Cancer Research Center (DKFZ), Im Neuenheimer Feld 280, 69120 Heidelberg, Germany. [2]Faculty of Biosciences, Heidelberg University, 69120 Heidelberg, Germany. [3]VIB-VUB Center for Structural Biology, Vlaams Instituut voor Biotechnologie, B-1050 Brussels, Belgium; [4]Brussels Center for Redox Biology, Vrije Universiteit Brussel, B-1050 Brussels, Belgium; [5]Structural Biology Brussels, Vrije Universiteit Brussel, B-1050 Brussels, Belgium

Correspondence and requests for materials should be addressed to T.P.D. (email: t.dick@dkfz.de)

Supplementary methods

Förster Resonance Energy Transfer (FRET) measurements. 4×10^4 HEK293 MSR cells per well were seeded into a 96-well plate. Cells were transfected the next day using Lipofectamine 2000 according to the manufacturer's instructions. 24 h after transfection, medium was replaced by pre-warmed DPBS and FRET was measured in a microplate reader (BMG Clariostar™). Emission was recorded at 530 nm, following excitation at 420 nm. The FRET signal was normalized to the Cerulean signal at 480 nm emission, following excitation at 420 nm.

Immunoprecipitation of endogenous STAT3. 3 µg of rabbit anti-STAT3 (Cell Signaling) antibody or rabbit IgG control antibody were bound to protein A-coated Dynabeads and then incubated overnight with 2 mg whole cell lysate from HEK293 MSR cells at 4°C. Following three washes with lysis buffer (1% Triton X-100 in TBS), the immunoprecipitates were eluted in 2X Laemmli buffer at 95°C and further subjected to immunoblotting.

Depletion of Prx2. The siRNA targeting Prx2 mRNA (5'-GAGAUCAUCGCGUUCAGCA-3') was transfected using Lipofectamine 3000 as per manufacturer's instructions. Scrambled siRNA (5'-GAAUGCUCAUGUUGAAUCA-3') was used as the negative control.

Quantitative Real-time PCR. RNA was isolated using the RNeasy kit (Qiagen). One microgram of RNA was used to synthesize cDNA using the iScript™ cDNA Synthesis Kit (Bio-Rad). 10 ng of cDNA were mixed with forward and reverse primers (0.5 µM each; see Supplementary Table S2) and the LightCycler® 480 SYBR Green I Master reaction mix (Roche). A LightCycler 480 (Roche) was used to perform the RT-PCR. The expression of the FOS gene was normalized to the expression of actin. Relative expression was calculated using the ΔΔCt method.

Phosphorylation of STAT3. Following serum starvation for 24 h, HEK293 MSR or U2OS cells were treated with 50 ng/mL IL-6/IL-6 receptor for the time periods indicated in Supp. Fig. 5e-f. Cells were lysed with 1% Triton X-100 in TBS supplemented with protease inhibitor (cOmplete™, Roche). Post nuclear lysates were subjected to reducing SDS-PAGE and immunoblot analysis.

Supplementary tables

Supplementary Table 1. Primers used for site-directed mutagenesis

	Name	Primer sequence (5'-3')
STAT3 and NTD	L78R Forward	CTGCAAGAGTCGAATGTTCGCTATCAGCACAATCTACG
	L78R Reverse	CGTAGATTGTGCTGATAGCGAACATTCGACTCTTGCAG
	W37A Forward	GGCAGTTTCTGGCCCCTGCGATTGAGAGTCAAGATTG
	W37A Reverse	CAATCTTGACTCTCAATCGCAGGGGCCAGAAACTGCC
AnxA2	C8S Forward	CTCCAAGCTGAGCTTGCTCAGGATTTCGTGAACAG
	C8S Reverse	CTGTTCACGAAATCCTGAGCAAGCTCAGCTTGGAG
	C132S Forward	CTGGTTGGTTCTGGAGCTGATGATCTCAATGAGAG
	C132S Reverse	CTCTCATTGAGATCATCAGCTCCAGAACCAACCAG

Supplementary Table 2. Primers used for qPCR

Name	Primer sequence (5'-3')
FOS Forward	AGGAGGGAGCTGACTGATACACT
FOS Reverse	TTTCCTTCTCCTTCAGCAGGTT
Actin Forward	CGTCACCAACTGGGACGACA
Actin Reverse	CTTCTCGCGGTTGGCCTTGG

a

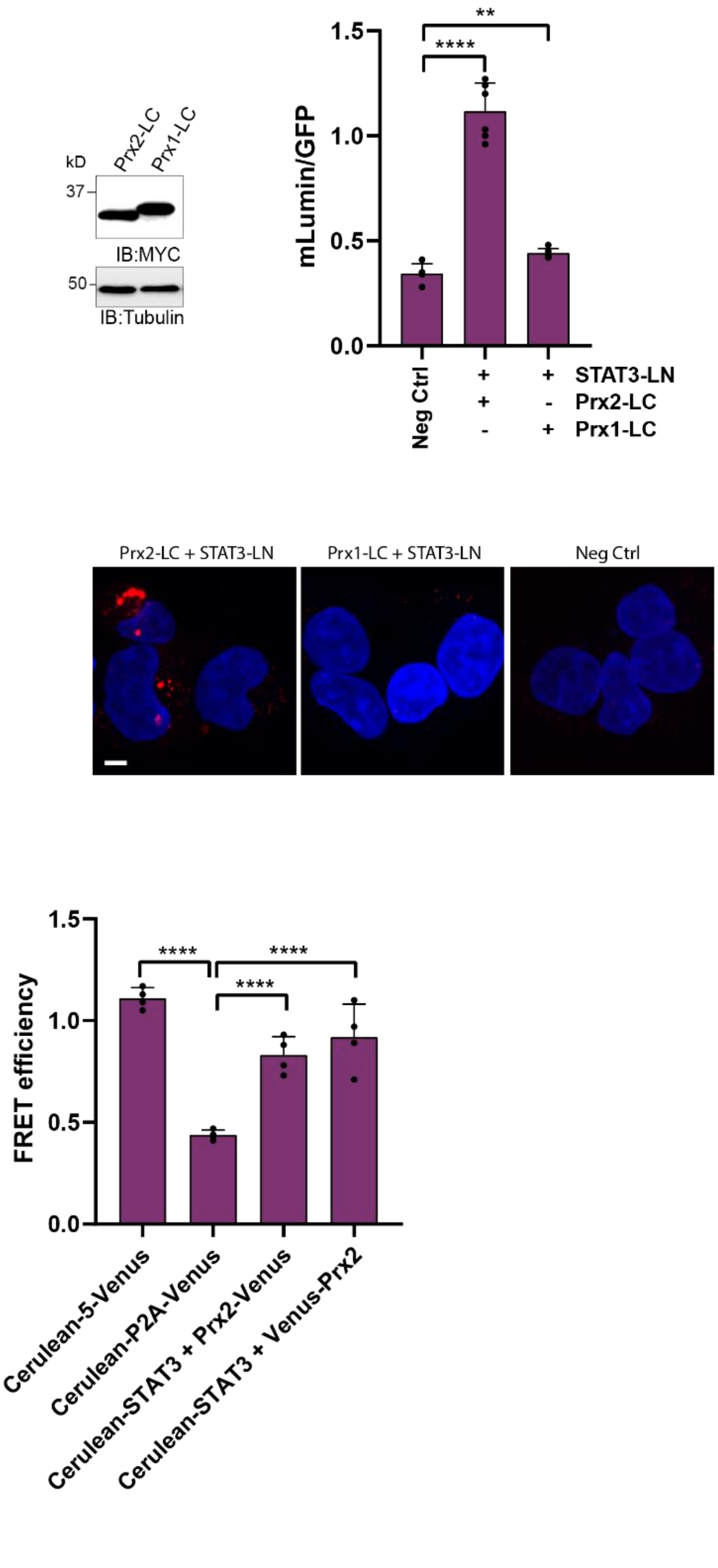
kD
Prx2-LC
Prx1-LC
37
IB:MYC
50
IB:Tubulin
1.5
1.0
0.5
0.0
mLumin/GFP
**
****
Neg Ctrl
+ + STAT3-LN
+ - Prx2-LC
- + Prx1-LC
b
Prx2-LC + STAT3-LN
Prx1-LC + STAT3-LN
Neg Ctrl
c
FRET efficiency
****
****
****
Cerulean-5-Venus
Cerulean-P2A-Venus
Cerulean-STAT3 + Prx2-Venus
Cerulean-STAT3 + Venus-Prx2

Supplementary Figure 1. STAT3 and Prx2 interact with each other

a The BiFC experiment (right panel) shows the same result as main Fig. 1a (bars 1-3). Here, we additionally show an anti-myc immunoblot (left panel) of the same cells to demonstrate the near-equal expression of Prx2-LC and Prx1-LC. Prx2-LC and Prx1-LC (both containing a myc-tag in the linker peptide) were transiently expressed in HEK293 MSR cells stably expressing STAT3-LN. The negative control ("Neg Ctrl") indicates co-expression of unfused LC and LN. Based on n = 6 independent experiments with n = 6 technical replicates each. Bar charts in this figure represent the mean ± SD. **p< 0.01; ****p< 0.0001; two-tailed unpaired t-test was used. IB: Immunoblot.

b Fluorescence complementation is localized to punctate structures. Confocal microscopy of HEK293 MSR cells co-transfected with STAT3-LN and Prx2-LC reveals mLumin fluorescence (red) in punctate structures (left panel), suggesting that complex formation is restricted to particular subcellular locations. Similar to the plate reader experiment (Fig. 1a and Suppl. Fig. 1a), the co-transfection of STAT3-LN and Prx1-LC yields little fluorescence complementation (middle panel). The negative control ('Neg Ctrl') shows co-transfection of unfused LN and LC, which do not show any significant complementation (right panel). Nuclei were stained with DAPI (blue). Scale bar 5 µm. The imaged cells are representative for n = 30-40 cells monitored in n = 2 independent experiments with n = 4 technical replicates each.

c FRET confirms the interaction of Prx2 and STAT3. HEK293 MSR cells were transfected with the indicated constructs. The Cerulean-5-Venus fusion protein was used as a positive control. The Cerulean-P2A-Venus fusion protein (which is subject to self-cleavage) was used as a negative control. Co-transfection of Cerulean-STAT3 with either Prx2-Venus or Venus-Prx2 led to a significant increase in FRET efficiency. Bars represent the mean (±SD) from n=4 independent experiments with n=6 technical replicates each. ****p< 0.0001.

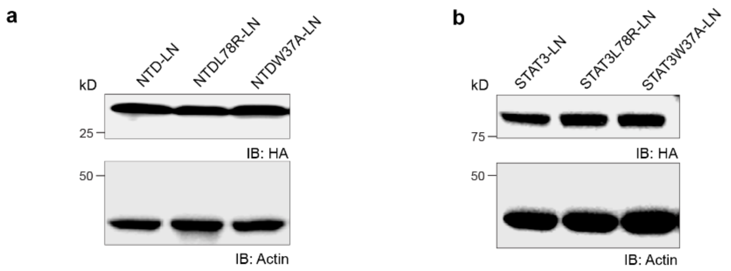

Supplementary Figure 2. Expression of single point mutants of NTD and STAT3

a The immunoblot confirms near-equal expression of wild type and mutant NTD domains for the experiment shown in main Fig. 2c. IB: Immunoblot.

b The immunoblot confirms near-equal expression of wild type and mutant STAT3 for the experiment shown in main Fig. 2d. IB: Immunoblot.

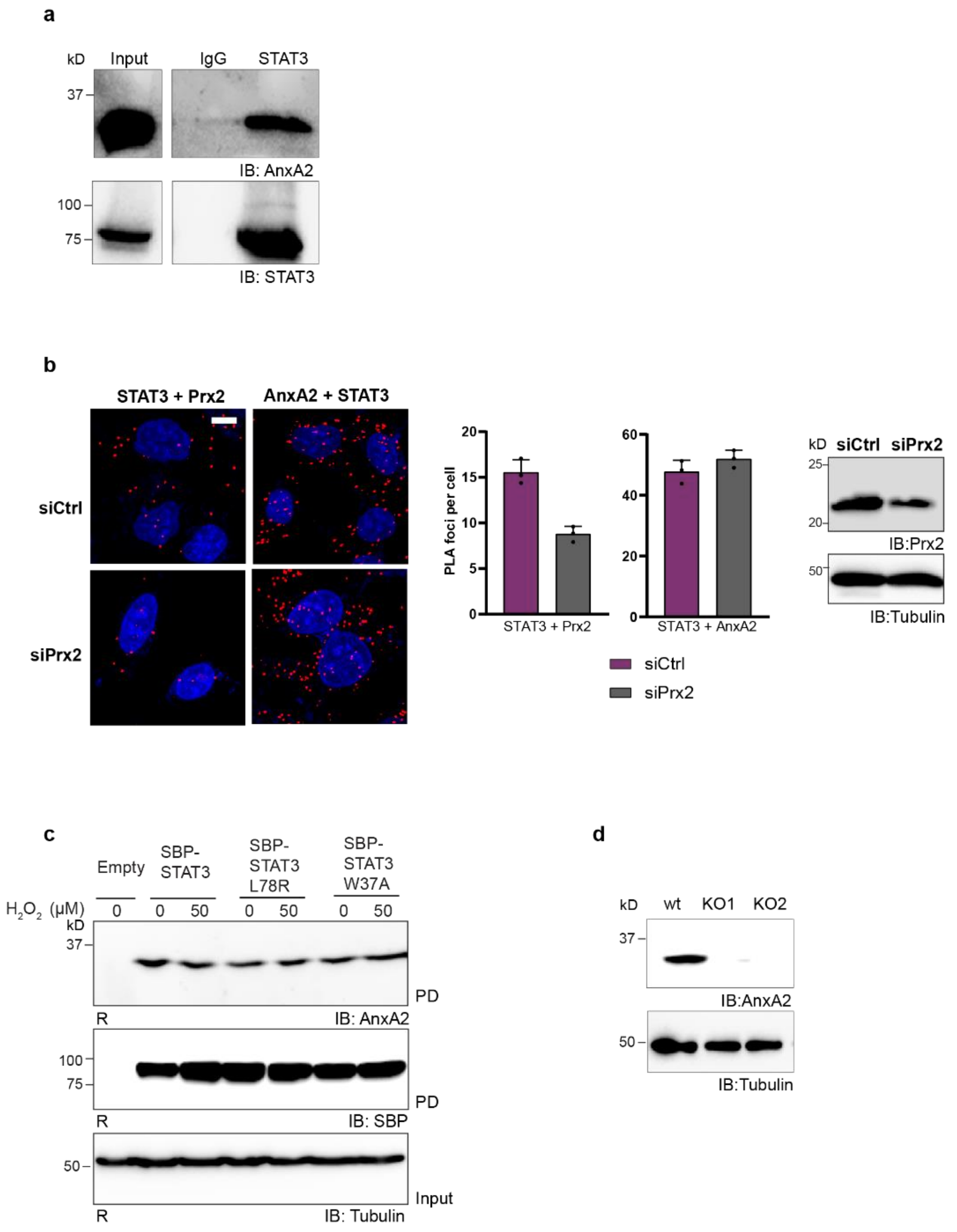
a
kD
Input
IgG
STAT3
37
IB: AnxA2
100
75
IB: STAT3
b
STAT3 + Prx2
AnxA2 + STAT3
siCtrl
siPrx2
PLA foci per cell
20
15
10
5
0
STAT3 + Prx2
60
40
20
0
STAT3 + AnxA2
siCtrl
siPrx2
kD
siCtrl
siPrx2
25
20
IB:Prx2
50
IB:Tubulin
c
Empty
SBP-STAT3
SBP-STAT3 L78R
SBP-STAT3 W37A
H2O2 (μM)
0
0
50
0
50
0
50
kD
37
PD
R
IB: AnxA2
100
75
PD
R
IB: SBP
50
Input
R
IB: Tubulin
d
kD
wt
KO1
KO2
37
IB:AnxA2
50
IB:Tubulin

Supplementary Figure 3. Interaction of AnxA2 with STAT3

a Endogenous AnxA2 is co-immunoprecipitated with endogenous STAT3. Endogenous STAT3 was immunoprecipitated from HEK293 MSR cell lysates, using isotype-matched non-specific antibody (IgG) as a control (lower panel). Immunoblotting demonstrates co-precipitation of AnxA2 (upper panel). The blot is representative of n = 2 independent experiments. IB: Immunoblot

b Depletion of Prx2 by siRNA does not compromise the AnxA2-STAT3 interaction as detected by PLA. Representative images show PLA foci in red and DAPI-stained nuclei in blue (left panels). Scale bar: 10 µm. The bar graphs (middle panel) show the PLA foci per cell in Prx2 depleted cells (siPrx2) relative to wild type cells (siCtrl) (n = 40-50 cells with n = 2 technical replicates). The interaction of Prx2 with STAT3 in cells with partial depletion of Prx2 was used as a control. The interaction of AnxA2 with STAT3 was not affected in Prx2 depleted cells. The Prx2 depletion efficiency (≈50%) is shown by immunoblotting (right panel). The result is representative of n = 3 independent experiments. Bars represent the mean (±SD). IB: Immunoblot.

c STAT3, STAT3(L78R) and STAT3(W37A) co-precipitate similar levels of AnxA2. The immunoblots are part of the experiment shown in main Fig. 2e. PD: Pulldown; IB: Immunoblot; R: Reducing gel electrophoresis.

d CRISPR/Cas9 mediated deletion of AnxA2 expression. The immunoblot confirms the abolition of AnxA2 protein expression in two independent HeLa clones generated by CRISPR/Cas9 (KO1 and KO2). These cells were used in main Fig. 3d. The blot is representative of n = 3 independent experiments. IB: Immunoblot.

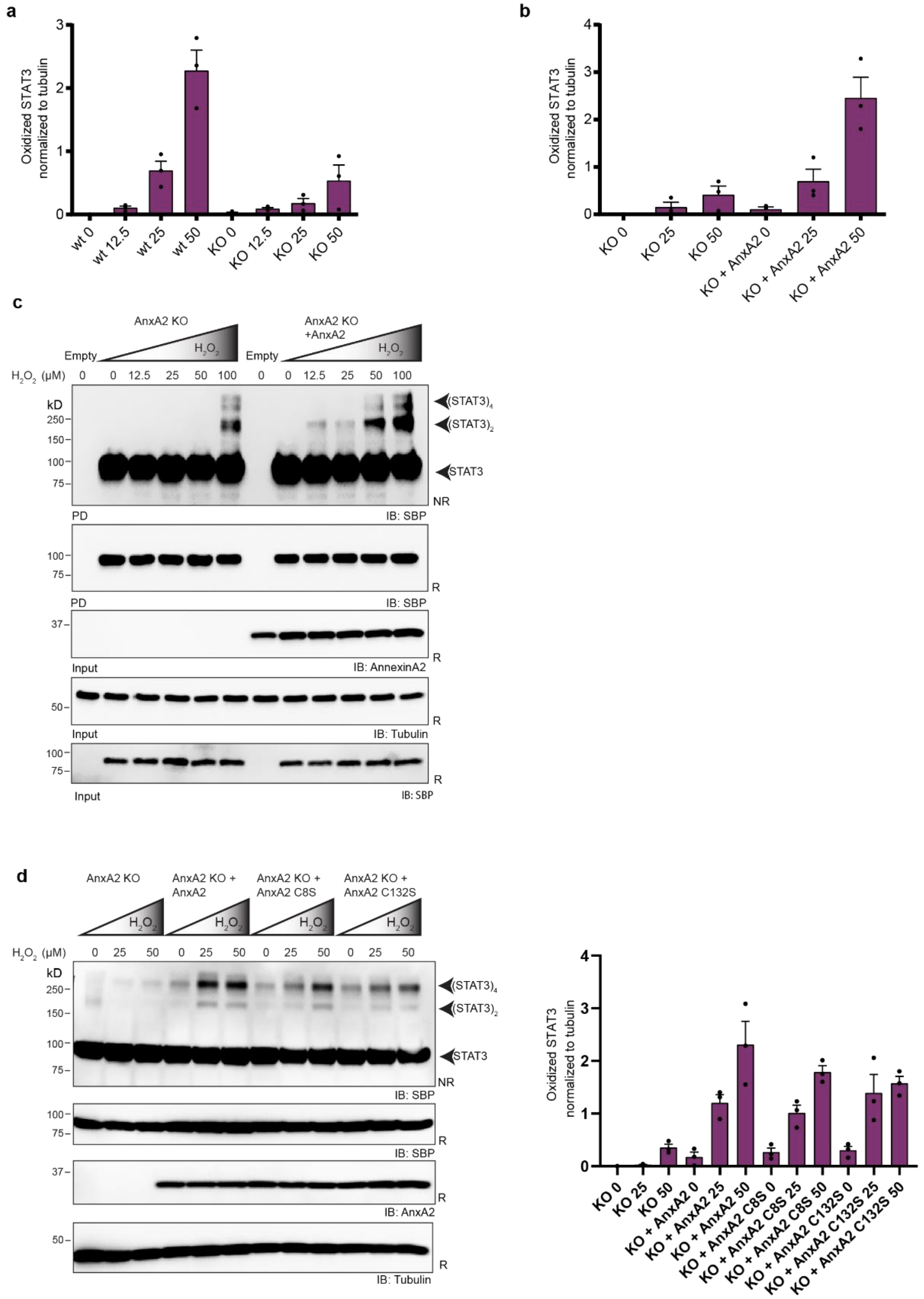
a
Oxidized STAT3 normalized to tubulin
wt 0
wt 12.5
wt 25
wt 50
KO 0
KO 12.5
KO 25
KO 50
b
Oxidized STAT3 normalized to tubulin
KO 0
KO 25
KO 50
KO + AnxA2 0
KO + AnxA2 25
KO + AnxA2 50
c
AnxA2 KO
AnxA2 KO +AnxA2
Empty
H_2O_2
H_2O_2 (μM)
0
0
12.5
25
50
100
kD
250
150
100
75
$(STAT3)_4$
$(STAT3)_2$
STAT3
NR
PD
IB: SBP
R
IB: AnnexinA2
Input
IB: Tubulin
d
AnxA2 KO
AnxA2 KO + AnxA2
AnxA2 KO + AnxA2 C8S
AnxA2 KO + AnxA2 C132S
IB: AnxA2
KO + AnxA2 C8S 0
KO + AnxA2 C8S 25
KO + AnxA2 C8S 50
KO + AnxA2 C132S 0
KO + AnxA2 C132S 25
KO + AnxA2 C132S 50

Supplementary Figure 4. STAT3 oxidation is dependent on the presence of AnxA2

a Quantification of oxidized STAT3 (covalent oligomers) normalized to Tubulin, relating to Fig. 4d. Bars show the mean (± SEM) of n = 3 biological replicates.

b Quantification of oxidized STAT3 (covalent oligomers) normalized to Tubulin, relating to Fig. 4e. Bars show the mean (± SEM) of n = 3 biological replicates.

c H_2O_2-induced STAT3 oxidation is largely rescued upon ectopic expression of AnxA2 in AnxA2 KO cells. Similar experiment as in main Fig. 4e but using additional concentrations of H_2O_2. PD: Pulldown; IB: Immunoblot; NR/R: Non-reducing/Reducing gel electrophoresis.

d The same experiment as shown in main Fig. 4e, but including the AnxA2(C8S) and AnxA2(C132S) mutants. The rescue of H_2O_2-induced STAT3 oxidation by ectopic expression of mutant AnxA2 is slightly less effective than rescue by wild type AnxA2 (left panel). Quantification of oxidized STAT3 (covalent oligomers) normalized to Tubulin (right panel). Bars show the mean (± SEM) of n = 3 biological replicates. IB: Immunoblot; NR/R: Non-reducing/Reducing gel electrophoresis. Source data is provided in the Source Data file.

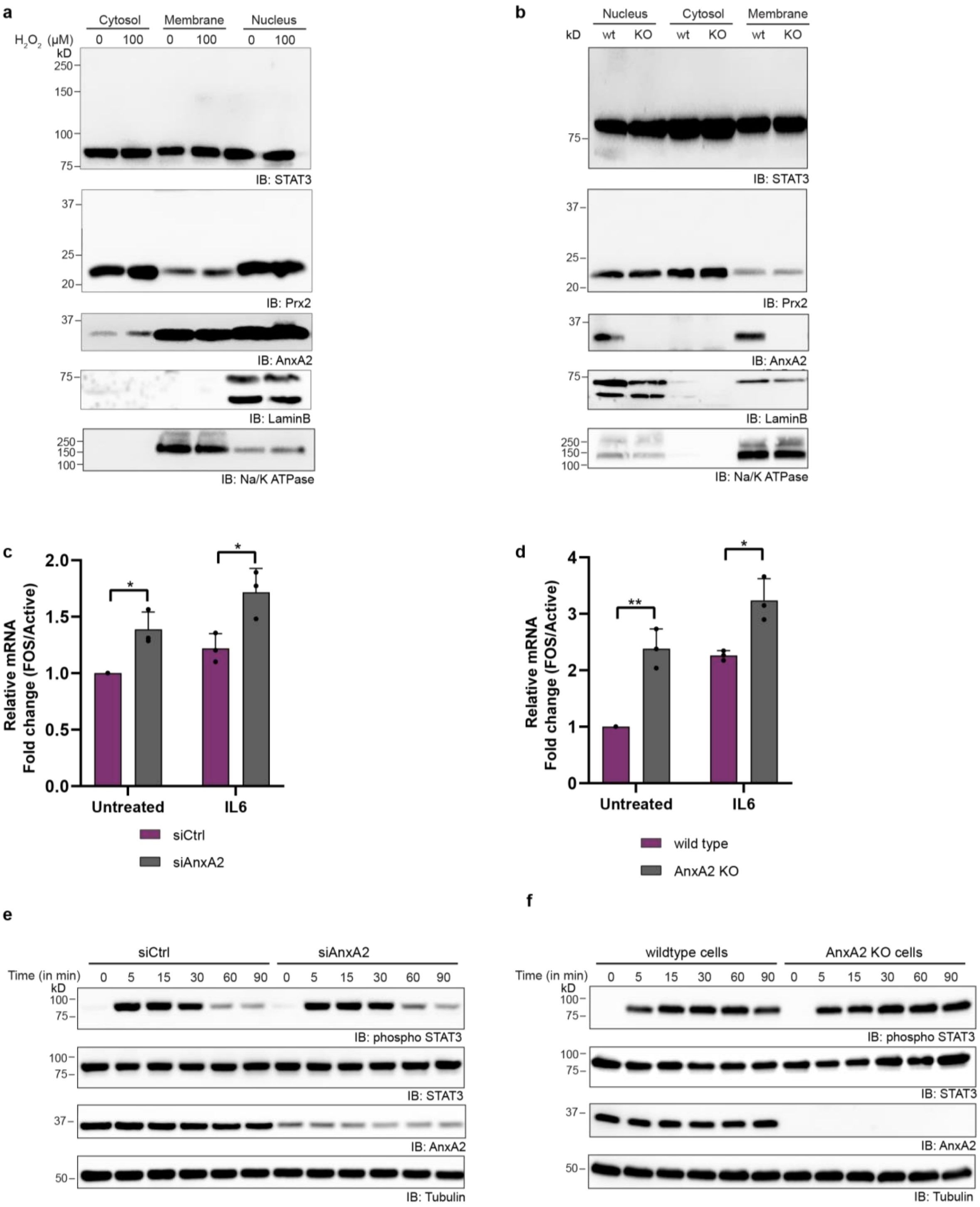
a
Cytosol
Membrane
Nucleus
H2O2 (µM)
0 100 0 100 0 100
kD
250
150
100
75
IB: STAT3
37
25
20
IB: Prx2
IB: AnxA2
IB: LaminB
IB: Na/K ATPase
b
Nucleus
Cytosol
Membrane
wt KO wt KO wt KO
IB: STAT3
IB: Prx2
IB: AnxA2
IB: LaminB
IB: Na/K ATPase
c
Relative mRNA
Fold change (FOS/Active)
Untreated
IL6
siCtrl
siAnxA2
d
Relative mRNA
Fold change (FOS/Active)
Untreated
IL6
wild type
AnxA2 KO
e
siCtrl
siAnxA2
Time (in min)
0 5 15 30 60 90 0 5 15 30 60 90
IB: phospho STAT3
IB: STAT3
IB: AnxA2
IB: Tubulin
f
wildtype cells
AnxA2 KO cells
Time (in min)
0 5 15 30 60 90 0 5 15 30 60 90
IB: phospho STAT3
IB: STAT3
IB: AnxA2
IB: Tubulin

Supplementary Figure 5. STAT3 oxidation occurs in association with membranes

a The experiment from main Fig. 5a, under reducing conditions, and with additional marker proteins. It confirms that the high-molecular weight bands seen in main Fig. 5a are disulfide-linked oligomers of STAT3 and Prx2, respectively. It also shows that AnxA2 is enriched in membrane and nuclear fractions relative to the cytosol. The blot is representative of n = 3 independent experiments. IB: Immunoblot.

b The experiment from main Fig. 5b, under reducing conditions, and with additional marker proteins. It confirms the deletion of AnxA2. The blot is representative of n = 3 independent experiments. IB: Immunoblot.

c The relative mRNA expression of the FOS gene as determined by qRT-PCR in U2OS cells increases upon depletion of AnxA2. Serum starved cells were treated with 50 ng/ml of IL6/IL6-R for 6 h. Actin mRNA expression was used for normalization. Bars represent the mean (±SD) of n = 3 independent experiments.

d The relative mRNA expression of the FOS gene as determined by qRT-PCR in HEK293 MSR cells increases upon depletion of AnxA2. Bars represent the mean (±SD) of n = 3 independent experiments.

e STAT3 Tyr-705 phosphorylation (phospho STAT3) is not affected by the depletion of AnxA2 in U2OS cells. Protein lysates from cells treated with 50 ng/mL IL6/IL6R for different time points were subjected to immunoblotting. IB: Immunoblot.

f STAT3 Tyr-705 phosphorylation (phospho STAT3) is not affected by the deletion of AnxA2 in HEK293 MSR cells. Protein lysates from cells treated with 50 ng/mL IL6/IL6R for different time points were subjected to immunoblotting. IB: Immunoblot.
