# Supplementary material for: A role for annexin A2 in scaffolding the peroxiredoxin 2–STAT3 redox relay complex
Source: Nat Commun. 2020 Sep 9;11:4512. doi: 10.1038/s41467-020-18324-9 (PMC7481202; doi:10.1038/s41467-020-18324-9)
Supplement: Supplementary file 2 — Reporting Summary [file 41467_2020_18324_MOESM2_ESM.pdf]

nature research

Corresponding author(s): Tobias P Dick

Last updated by author(s): Aug 4, 2020

Reporting Summary

Nature Research wishes to improve the reproducibility of the work that we publish. This form provides structure for consistency and transparency in reporting. For further information on Nature Research policies, see our Editorial Policies and the Editorial Policy Checklist.

Statistics

For all statistical analyses, confirm that the following items are present in the figure legend, table legend, main text, or Methods section.

n/a	Confirmed	
☐	☒	The exact sample size (*n*) for each experimental group/condition, given as a discrete number and unit of measurement
☐	☒	A statement on whether measurements were taken from distinct samples or whether the same sample was measured repeatedly
☐	☒	The statistical test(s) used AND whether they are one- or two-sided<br>*Only common tests should be described solely by name; describe more complex techniques in the Methods section.*
☒	☐	A description of all covariates tested
☒	☐	A description of any assumptions or corrections, such as tests of normality and adjustment for multiple comparisons
☐	☒	A full description of the statistical parameters including central tendency (e.g. means) or other basic estimates (e.g. regression coefficient) AND variation (e.g. standard deviation) or associated estimates of uncertainty (e.g. confidence intervals)
☐	☒	For null hypothesis testing, the test statistic (e.g. *F*, *t*, *r*) with confidence intervals, effect sizes, degrees of freedom and *P* value noted<br>*Give P values as exact values whenever suitable.*
☒	☐	For Bayesian analysis, information on the choice of priors and Markov chain Monte Carlo settings
☒	☐	For hierarchical and complex designs, identification of the appropriate level for tests and full reporting of outcomes
☒	☐	Estimates of effect sizes (e.g. Cohen's *d*, Pearson's *r*), indicating how they were calculated

Our web collection on statistics for biologists contains articles on many of the points above.

Software and code

Policy information about availability of computer code

Data collection	No software was used for data collection
Data analysis	GraphPad Prism 8, ImageJ and Adobe Illustrator CS6 were used for figure analysis and preparation.<br>NEBuilder Assembly Tool was used for primer design for Gibson assembly.

For manuscripts utilizing custom algorithms or software that are central to the research but not yet described in published literature, software must be made available to editors and reviewers. We strongly encourage code deposition in a community repository (e.g. GitHub). See the Nature Research guidelines for submitting code & software for further information.

Data

Policy information about availability of data

All manuscripts must include a data availability statement. This statement should provide the following information, where applicable:

- Accession codes, unique identifiers, or web links for publicly available datasets
- A list of figures that have associated raw data
- A description of any restrictions on data availability

All data generated or analysed during this study are included in this published article (and its supplementary information files).
The BioGRID database (https://thebiogrid.org/) was utilized to identify annexin A2 as the common interaction partner of Prx2 and STAT3.

Field-specific reporting

Please select the one below that is the best fit for your research. If you are not sure, read the appropriate sections before making your selection.

- [x] Life sciences
- [ ] Behavioural & social sciences
- [ ] Ecological, evolutionary & environmental sciences

For a reference copy of the document with all sections, see nature.com/documents/nr-reporting-summary-flat.pdf

Life sciences study design

All studies must disclose on these points even when the disclosure is negative.

Sample size	Group size for original data created here was based on previous experience. No statistical method was used to predetermine sample size.
Data exclusions	No data were excluded from analyses
Replication	All experiments were repeated at least 3 times (biological replicates)
Randomization	No randomization method was used.
Blinding	Data collection and Analysis were not blinded.

Reporting for specific materials, systems and methods

We require information from authors about some types of materials, experimental systems and methods used in many studies. Here, indicate whether each material, system or method listed is relevant to your study. If you are not sure if a list item applies to your research, read the appropriate section before selecting a response.

Materials & experimental systems

n/a	Involved in the study	
	x	Antibodies
	x	Eukaryotic cell lines
x		Palaeontology and archaeology
x		Animals and other organisms
x		Human research participants
x		Clinical data
x		Dual use research of concern

Methods

n/a	Involved in the study	
x		ChIP-seq
x		Flow cytometry
x		MRI-based neuroimaging

Antibodies

Antibodies used

The primary antibodies used in the study are listed here:
mouse anti-SBP (Santa Cruz, sc101595, clone SB19-C4, lot no. L1015)
rabbit anti-tubulin (Cell Signaling, 2128, clone 9F3, lot no. 7)
mouse anti-actin (Sigma, A5441, clone AC15, lot no. 127M4866V)
rabbit anti-HA tag (Cell Signaling, 3724, clone C29F4, lot no. 5)
rabbit anti-MYC tag (Cell Signaling, 2278, clone 71D10, lot no. 5)
rabbit anti-STAT3 (Cell Signaling, 12640, clone D3Z2G, lot no. 4)
mouse anti STAT3 (Cell Signaling, 9139, clone 124H6, lot no. 12)
rabbit anti-Prx2 (abcam, 109367, clone EPR5154, lot no. GR3257260-2)
mouse anti-Prx2 (Thermo Scientific, LF-MA0144, clone 1E8, lot no. TH2621314)
mouse anti-Annexin A2 (Santa Cruz, sc47696, clone 3D5, lot no. LZ717)
rabbit anti-Annexin A2 (Cell Signaling, 8235, clone D1162 , lot no. 2)
mouse anti-S100A10 (Cell Signaling, 5529, clone 4E7E10, lot no. 1)
goat anti-lamin B (Santa Cruz, sc6217, clone M-20, lot no. B1116)
mouse anti-gp130 (Santa Cruz, sc376280, clone E-8, lot no. J0917)
mouse anti-Na+/K+ ATPse (Santa Cruz, sc48345, clone H-3, lot no. K1219)
mouse anti-STAT3 phospho (Tyr705) (Cell Signaling, 9138, clone 3E2, lot no. 5)

The following secondary antibodies were used with 1:10000 dilution for western blotting:
Anti-goat (Santa Cruz, sc2020)
Anti-mouse (Jackson immunoResearch, 115-035-146)
Anti.rabbit (Jackson ImmunoResearch, 111-035-144)

Validation	Validation statement for each primary antibody is provided on the manufacturer's website.<br>Validation of anti-AnnexinA2 antibodies was performed through immunoblotting of lysates from annexinA2 KO cells (as shown in the manuscript).<br>Validation of anti-Prx2 antibodies was performed through immunoblotting of lysates of cells with or without transfection with siPrx2 (as shown in the manuscript) and also by additional control experiments (Prx2 KO cells).

Eukaryotic cell lines

Policy information about cell lines

Cell line source(s)	HEK293 MSR (GripTite™) cells were purchased from Thermo Fisher<br>HeLa, U2OS and Phoenix Ampho cells were purchased from ATCC
Authentication	The Multiplex human Cell Line Authentication test (MCA) was used to authenticate cell lines as described on www.multiplexion.de
Mycoplasma contamination	All cell lines were repeatedly tested negative for mycoplasma contamination as stated in Material and Methods
Commonly misidentified lines<br>(See ICLAC register)	No commonly misidentified lines were used
